# Association between neurosarcoidosis with autonomic dysfunction and anti-ganglionic acetylcholine receptor antibodies

**DOI:** 10.1007/s00415-021-10551-4

**Published:** 2021-04-21

**Authors:** Makoto Oishi, Akihiro Mukaino, Misako Kunii, Asami Saito, Yukimasa Arita, Haruki Koike, Osamu Higuchi, Yasuhiro Maeda, Norio Abiru, Naohiro Yamaguchi, Hiroaki Kawano, Eiko Tsuiki, Tomonori Tanaka, Hidenori Matsuo, Masahisa Katsuno, Fumiaki Tanaka, Akira Tsujino, Shunya Nakane

**Affiliations:** 1 Department of Neurology and Strokology, Nagasaki University Hospital, Nagasaki, Japan; 2 Department of Molecular Neurology and Therapeutics, Kumamoto University Hospital, 1-1-1, Honjo, Chuo-ku, Kumamoto, 860-8556 Japan; 3 Department of Neurology and Stroke Medicine, Yokohama City University Graduate School of Medicine, Yokohama, Japan; 4 Department of Neurology, Matsuyama Red Cross Hospital, Ehime, Japan; 5 Department of Neurology, Nagoya University Graduate School of Medicine, Nagoya, Japan; 6 Department of Clinical Research, Nagasaki Kawatana Medical Center, Nagasaki, Japan; 7 Department of Endocrinology and Metabolism, Unit of Advanced Preventive Medical Sciences, Division of Advanced Preventive Medical Sciences, Nagasaki University Graduate School of Biomedical Sciences, Nagasaki, Japan; 8 Department of Psychiatry, Nagasaki University Hospital, Nagasaki, Japan; 9 Department of Cardiology, Nagasaki University Hospital, Nagasaki, Japan; 10 Department of Ophthalmology, Nagasaki University Hospital, Nagasaki, Japan; 11 Department of Pathology, Nagasaki University Hospital, Nagasaki, Japan

**Keywords:** Neurosarcoidosis, Autonomic dysfunction, Anti-ganglionic acetylcholine receptor antibodies, Small fiber neuropathy

## Abstract

**Objective:**

To determine whether autonomic dysfunction in neurosarcoidosis is associated with anti-ganglionic acetylcholine receptor (gAChR) antibodies, which are detected in autoimmune autonomic ganglionopathy.

**Methods:**

We retrospectively extracted cases of sarcoidosis from 1787 serum samples of 1,381 patients between 2012 and 2018. Anti-gAChR antibodies against the α3 and β4 subunit were measured by luciferase immunoprecipitation to confirm the clinical features of each case. We summarized literature reviews of neurosarcoidosis with severe dysautonomia to identify relevant clinical features and outcomes.

**Results:**

We extracted three new cases of neurosarcoidosis with severe dysautonomia, among which two were positive for anti-gAChR antibodies: Case 1 was positive for antibodies against the β4 subunit, and Case 2 was positive for antibodies against both the α3 and β4 subunits. We reviewed the cases of 15 patients with neurosarcoidosis and severe dysautonomia, including the three cases presented herein. Orthostatic hypotension and orthostatic intolerance were the most common symptoms. Among the various types of neuropathy, small fiber neuropathy (SFN) was the most prevalent, with seven of nine cases exhibiting definite SFN. Six of eight cases had impaired postganglionic fibers, of which the present three cases revealed abnormality of ^123^I-MIBG myocardial scintigraphy. Of the 11 cases, 10 were responsive to immunotherapy, except one seropositive case (Case 2).

**Conclusions:**

The presence of gAChR antibodies may constitute one of the mechanisms by which dysautonomia arises in neurosarcoidosis.

## Introduction

Sarcoidosis presents with multiple non-caseating granulomas throughout the body, and neurological symptoms are thought to occur in 5–13% of the patients [1, 2]. Approximately 15% of patients with neurosarcoidosis develop peripheral neuropathy [3]. Small fiber neuropathy (SFN), characterized by sensory disturbance and autonomic failure due to damage to the myelinated Aδ and unmyelinated C fibers, is particularly common and has been reported in 44% of patients with sarcoidosis, potentially decreasing their quality of life [4–6].

Autoimmune autonomic ganglionopathy (AAG) is a rare disease characterized by various autonomic symptoms. The ganglionic neuronal nicotinic acetylcholine receptor (gAChR), consisting of two α3 and three β4 subunits, mediates fast synaptic transmission in all peripheral autonomic ganglia in the autonomic nervous system [7]. Antibodies against gAChR are detected in approximately 50% of patients with AAG. Although the clinical features of neurosarcoidosis with dysautonomia are similar to those of AAG, the exact mechanism by which these symptoms arise remains unclear.

Herein, we aimed to elucidate the relationship between neurosarcoidosis with autonomic dysfunction and the presence of anti-gAChR antibodies.

## Methods

### Patients

We examined 1,787 serum samples of 1,381 patients from teaching and general hospitals throughout Japan between January 2012 and August 2018. We detected serum gAChRα3 and β4 antibodies using the Luciferase Immunoprecipitation System assay and retrospectively identified cases that fulfilled the diagnostic guidelines for sarcoidosis [8, 9]. In the present study, antibody levels were expressed as an antibody index (AI), which was calculated as follows: AI = (measured value in the serum sample [in relative luminescence units (RLU)])/(cut-off value [in RLU]). The normal AI value, established based on data from healthy individuals, was < 1.0. We used the criteria for SFN proposed by Lacomis et al. [10], who stated that the diagnosis of SFN consists of three components: (1) symptoms of peripheral paresthesia that are typically painful, (2) specialized electrodiagnostic testing (normal nerve conduction studies and electromyogram), and (3) pathological findings [decreased intra-epidermal nerve fiber density (IENFD)]; these criteria are used to classify SFN as possible (one item positive), probable (two items positive), or definite (three items positive). Clinical data were obtained by reviewing the case records at each hospital.

All patients provided written informed consent for the storage and use of their serum and clinical information for research purposes. The study was approved by the Human Ethics Committees at the Nagasaki Kawatana Medical Center and Kumamoto University Hospital (Japan) (approval number 2011–21 and 1281, respectively).

### Histological analysis of skin biopsy

A 3-mm punch biopsy was performed under local anesthesia (1% lidocaine) in the right lower abdomen and medial surface of the right lower leg. Then, 50-μm thick sections were immunostained using anti-human PGP 9.5 rabbit polyclonal rabbit antibody (Bio-Rad formerly AbDserotec #7863–0504, Hercules, CA), and horseradish peroxidase-conjugated anti-rabbit polyclonal immunoglobulin G secondary antibody (#424,144, NICHIREI BIOSCIENCES INC, Tokyo, Japan). We counted the number of small fibers in the dermis.

### Literature review

A systematic review of the literature was conducted following the Preferred Reporting Items for Systematic Reviews and Meta-Analyses (PRISMA) reporting guidelines [11]. We also used Google Scholar and PubMed for our search targeting relevant peer-reviewed articles that were published between September 1985 and March 2020 using the following medical subject heading terms: “sarcoidosis”, “neurosarcoidosis”, “autonomic”, and “small fiber neuropathy”. The reference lists were found in relevant papers and textbooks manually. We extracted and tabulated data, including age at the onset of sarcoidosis, sex, mode of onset, autonomic symptoms, site of lesion in dysautonomia, immunotherapy and clinical outcome. We compared the frequency of autonomic dysfunction among cases of neurosarcoidosis with severe dysautonomia and anti-gAChR antibody-positive AAG.

### Statistical analysis

A commercially available statistical software (SigmaPlot®; SPSS, Inc., Chicago, IL, USA) was used to analyze the data. We compared the prevalence of symptoms and associated data between patients with neurosarcoidosis with dysautonomia and those with anti-gAChR antibody-positive AAG; each of the analysis was assumed to be independent. Normally distributed data in both groups were analyzed by the Student’s t-test for continuous variables (age at diagnosis) and by the Chi-squared test for categorical variables. For all analyses, P < 0.05 was considered to reflect statistically significant differences between groups.

## Results

### General sample characteristics

Among 1,381 patients, 179 gAChR antibody-positive patients had comorbid diseases, of whom 53 patients (30%) presented with other autoimmune diseases, including Sjögren's syndrome in 20 patients and Hashimoto's disease in 13 patients; 19 (11%) presented with tumors [ovarian (*n* = 5), lung (*n* = 5), gastric (*n* = 3), prostate (*n* = 2), maxillary sinus (*n* = 1), and mediastinal (*n* = 1) tumors; thymoma (*n* = 1); and seminoma (*n* = 1)] [12].

### Case presentation

We examined three new cases of neurosarcoidosis presenting with severe autonomic disturbance.

#### Case 1

**Fig. 1:**
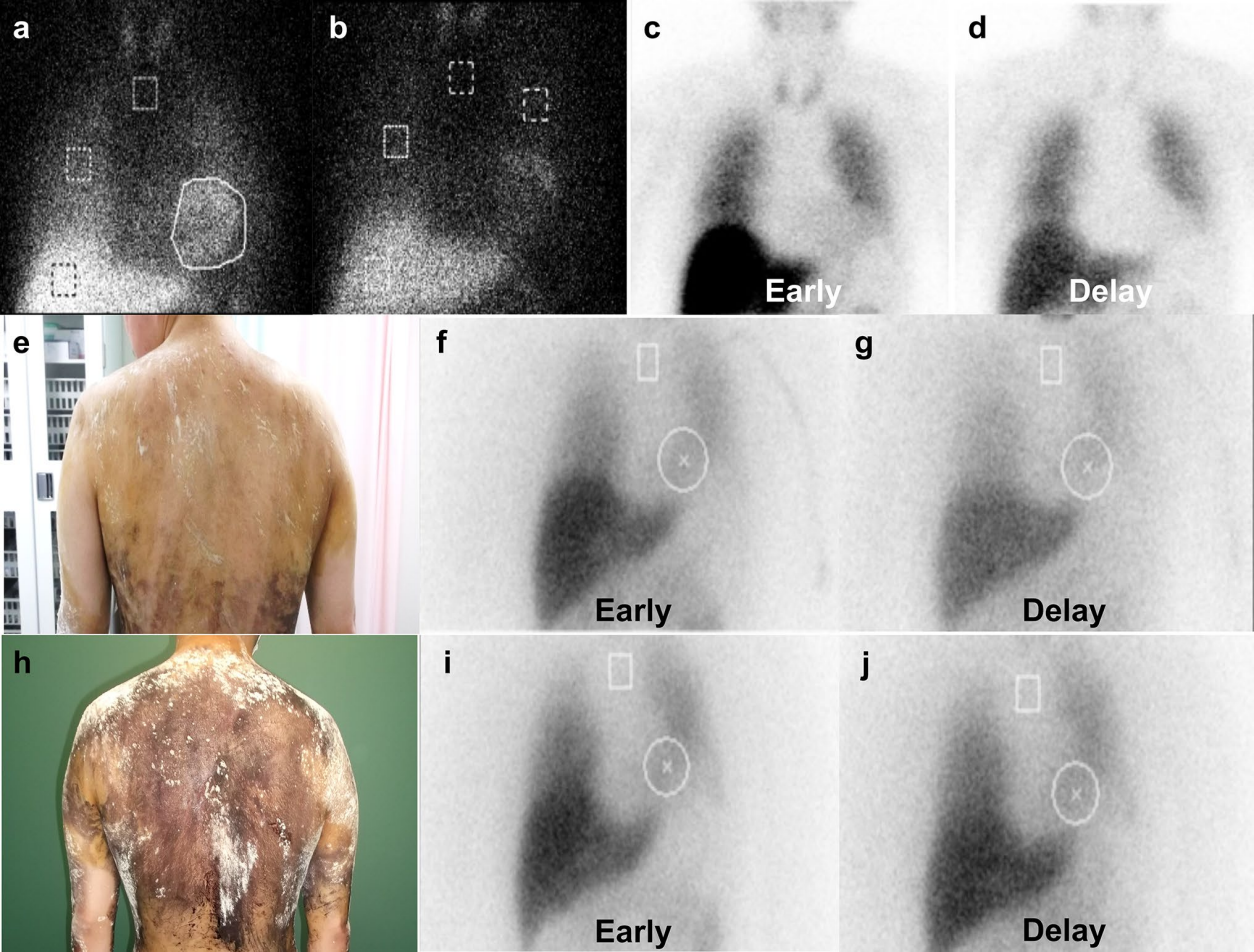
^123^I-MIBG myocardial scintigraphy and thermoregulatory sweating test. **a**–**d**, **f**, **g**, **i**, **j** Iodine-123 and meta-iodobenzylguanidine myocardial scintigraphy of Cases 1–3. Note the reduced heart-to-mediastinum (H/M) ratio: a early 1.94, **b** delayed 1.70 in Case 1; **c** early 1.53, **d** delayed 1.44 in Case 2; and **f**, **g** pre-immunotherapy: **f** early 1.49, **g** delayed 1.39 and increased washout rate (46.2%) in Case 3. **i**, **j** Post-immunotherapy. Note the slight improvements in the H/M ratio: **i** early 1.66, **j** delayed 1.63 and washout rate (28.5%). **e**, **h** Thermoregulatory sweating test in Case 3. **e** Pre-immunotherapy. Note the reduced sweating. **h** Post-immunotherapy. Note that generalized sweating was apparent

A 64-year-old man was admitted to the hospital due to orthostatic dizziness and recurrent syncope for 3 months. He had been diagnosed with pulmonary sarcoidosis 2 years prior to admission and treated with oral prednisolone (10 mg per day). He exhibited autonomic symptoms including orthostatic hypotension, orthostatic intolerance, xerophthalmia, constipation, and impotence. The serum levels of angiotensin-converting enzyme (ACE) were elevated (40.4 U/L [normal range 21.4 U/L]). The coefficient of variation of R–R intervals (CV R–R) was low (1.09% at rest). Iodine-123 (^123^I) and meta-iodobenzylguanidine (^123^I-MIBG) myocardial scintigraphy revealed a reduced heart-to-mediastinum (H/M) ratio (early 1.94, delayed 1.70 [normal range ≥ 2.20, respectively]) and increased washout rate (57.71%) (Fig. 1a, b). Single photon emission computed tomography with ioflupane I-123 injection (DaTscan™) was normal. The patient was positive for anti-gAChR antibodies against β4 (AI: 1.012).

#### Case 2

**Fig. 2:**
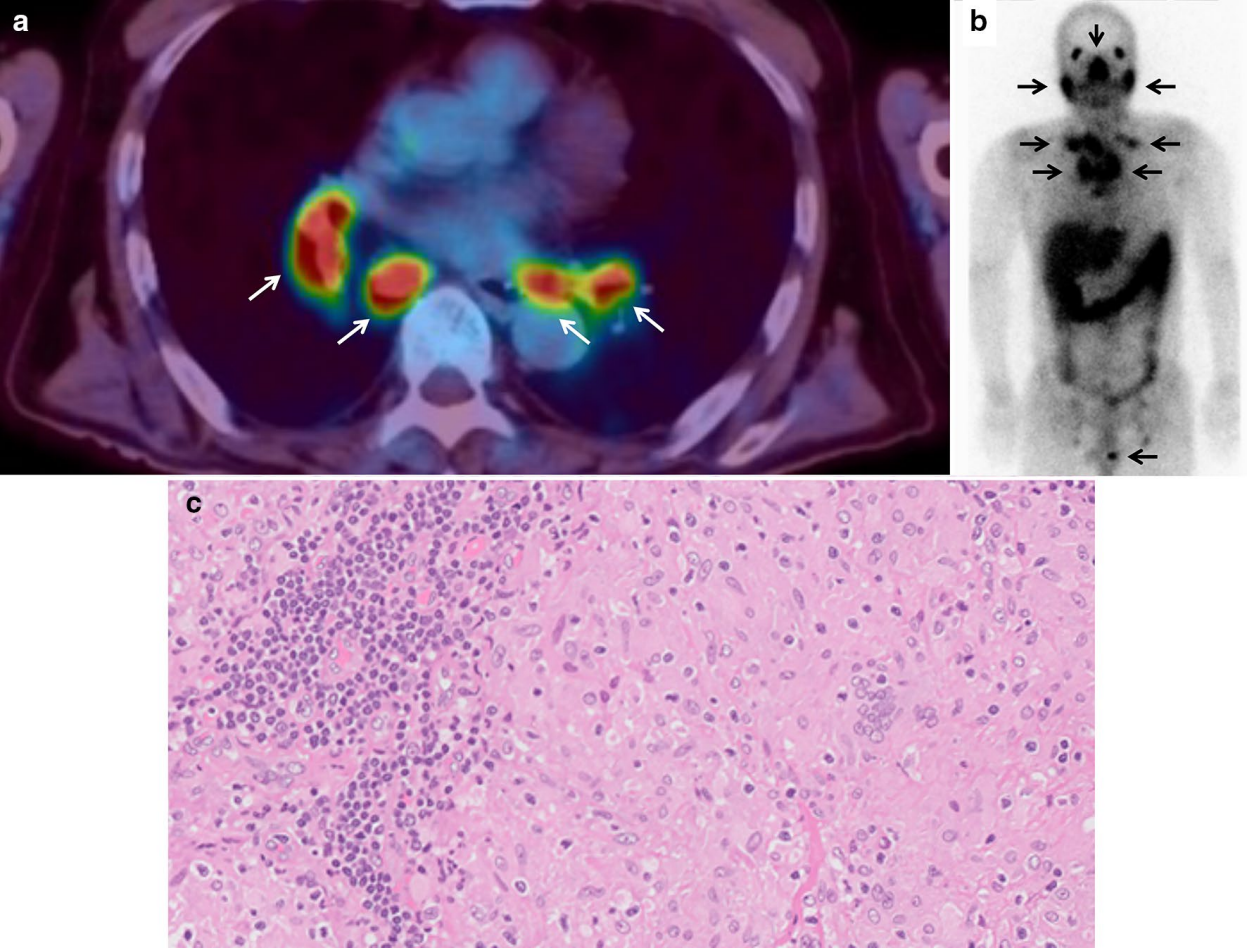
Image and pathological findings of patients. **a** Fluorodeoxyglucose (FDG)-positron emission tomography of Case 2. Accumulation of FDG in the hilar and mediastinal lymph nodes. **b** Gallium-67 scintigraphy. Accumulations in the lacrimal and salivary glands, para-aortic and left groin lymph nodes, and left testis. **c** Inguinal lymph node biopsy. Non-caseating granuloma (hematoxylin–eosin staining, scale bar = 500 μm)

**Fig. 3:**
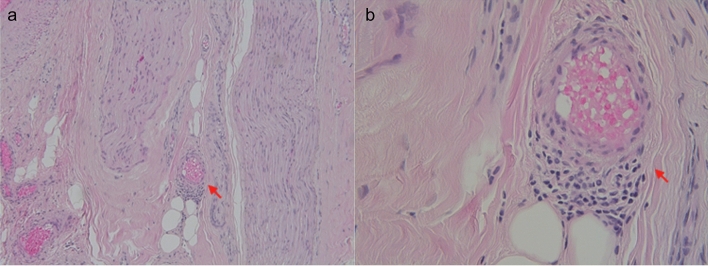
Pathology of the sural nerve biopsy and skin biopsy of the abdomen and leg. **a**, **b** Sural nerve biopsy. Inflammatory cellular infiltration around epineurial small vessels. Arrow: inflammatory cell infiltration

A 69-year-old man was admitted to the hospital due to recurrent syncope and urinary retention for 2 years. He had been experiencing constipation for 10 years. A year prior to presentation, he had been prescribed midodrine hydrochloride, amezinium metilsulfate, and droxidopa, which had improved syncope; however, orthostatic intolerance persisted. He was diagnosed with pulmonary, skin, and ocular sarcoidosis. On admission, he exhibited various autonomic symptoms, including orthostatic hypotension, supine hypertension, orthostatic intolerance (dizziness, syncope), alternating loose stools, and constipation, as well as urinary retention. He also complained of a burning sensation from the umbilicus to the lateral side of his lower leg and great toe. Fluorodeoxyglucose (FDG)-positron emission tomography demonstrated the accumulation of FDG in the hilar and mediastinal lymph nodes (Fig. 2a). ^123^I-MIBG myocardial scintigraphy revealed a reduced H/M ratio (early 1.53, delayed 1.44) and increased washout rate (34.4%) (Fig. 1c, d). Sympathetic skin response induced by stimulation of the median nerve demonstrated a low amplitude. The patient was positive for anti-gAChR antibodies against both α3 and β4 subunits (AI 1.787 and 1.118, respectively). Sural nerve biopsies revealed inflammatory cellular infiltration around the epineurial small vessels (Fig. 3a, b) and intact myelinated fibers. The patient was treated with two courses of steroid pulse therapy followed by oral administration of 40 mg prednisolone per day. Although ocular sarcoidosis improved, autonomic symptoms did not improve. Due to glaucoma, his dose of prednisolone was tapered by 5 mg per day over a month to a final dose of 5 mg. Despite administration of intravenous immunoglobulin (IVIg), autonomic symptoms did not improve after 3 years.

#### Case 3

A 27-year-old man with a history of type 1 diabetes mellitus and extensive dysautonomia was admitted to the hospital. He exhibited diabetic retinopathy. Upon admission, physical examination revealed sinus tachycardia with a pulse rate of 109 beats/min. There were palpable enlargements of the lymph nodes on the left side of the neck and groin, and a nodule in the left testis. Neurological examination revealed paresthesia in the distal extremities and absent Achilles-tendon reflexes. The patient exhibited various autonomic symptoms, including orthostatic intolerance, dry eyes and mouth, dry skin, alternating loose stools and constipation, neurogenic bladder, and erectile dysfunction. The serum levels of calcium and ACE were normal, and glycated hemoglobin was moderately elevated (7.5% [normal range 6.2%]). Antibodies against gAChR were absent. The total cerebrospinal fluid protein levels were slightly elevated (52 mg/dL [normal range 40 mg/dl]); however, cell count, glucose, ACE, and immunoglobulin G index were normal. Nerve conduction examinations yielded no significant findings. Head-up tilt test results were consistent with neurally mediated syncope; systolic blood pressure dropped from 100 to 80 mmHg under isoproterenol provocation. The CV R–R was extremely low (1.18% and 1.11% at rest and deep inspiration, respectively). A thermoregulatory sweat test revealed reduced sweating (Fig. 1e). ^123^I-MIBG myocardial scintigraphy revealed a reduced H/M ratio (early 1.49, delayed 1.39) and increased washout rate (46.2%; Fig. 1f, g). Contrast-enhanced computed tomography demonstrated swollen hilar, mediastinal, supraclavicular fossa, cervical, axillary, lesser curvature, para-aortic, posterior pancreatico-duodenal, and inguinal lymph nodes; splenomegaly; multiple nodular lesions in the spleen; and an infiltrative, nodular shadow in the right lung. Gallium-67 scintigraphy revealed accumulations in the lacrimal and salivary glands, para-aortic and left groin lymph nodes, and left testis (Fig. 2b). Furthermore, gadolinium-enhanced brain magnetic resonance imaging revealed enlargement of the pituitary gland and hypothalamus. Inguinal lymph node biopsy revealed a non-caseating granuloma (Fig. 2c). We began oral administration of 60 mg prednisolone per day. Surprisingly, the patient’s autonomic symptoms improved dramatically. His constipation and light-headedness resolved; however, he continued to exhibit mild orthostatic tachycardia. His skin became less dry, and generalized sweating was apparent (Fig. 1h). ^123^I-MIBG myocardial scintigraphy revealed slight improvements in the H/M ratio (early 1.66, delayed 1.63) and washout rate (28.5%; Fig. 1i, j). Decreasing the dosage of steroids did not cause a recurrence of symptoms; therefore, prednisolone treatment was reduced by 15 mg per day after 9 months.

### Histological analysis of skin biopsy

In Case 2, the skin biopsy from the right lower abdomen and medial surface of the right lower leg demonstrated a decrease in the dermal nerve fiber density (Supplementary Figure).

### Systematic literature review

**Fig. 4:**
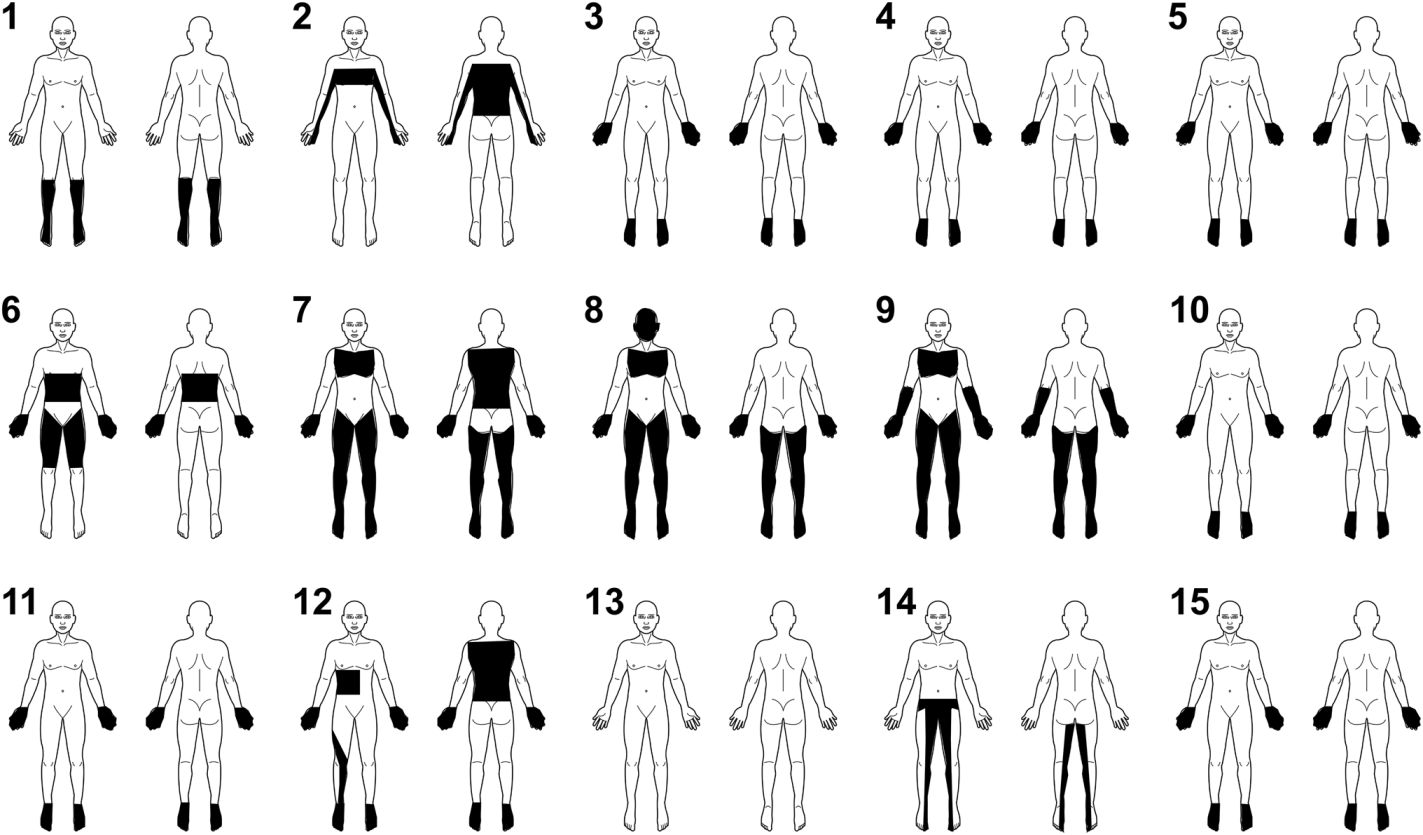
Body diagrams representing the regional distribution of sensory symptoms in 15 patients. Patient 1 = the legs and feet, 2 = the C8-Th6 level and lower back; 3–5, 10, 11, 15 = the hands and feet; 6 = the hands, Th4-11 level, and anterior region of thigh; 7 = the hands, chest, lower back, legs, and feet; 8 = the face, hands, chest, legs, and feet; 9 = the hands, forearms, chest, legs, and feet; 12 = the hands, right Th5-11, back, right L4, and feet; 14 = the umbilicus, lateral side of lower leg, and great toe

We obtained data for 15 cases of sarcoidosis with severe dysautonomia, including the three cases described above (designated as patients 13, 14, 15, respectively, in Table 1) [13–21]. Out of the nine patients with SFN, seven had definite SFN (patients 3–5, 7–9, 12) and two (patients 14, 15) had probable SFN. The mean age at onset of sarcoidosis and dysautonomia was 47.4 ± 14.0 and 48.3 ± 13.3 years, respectively. All patients except patient 13 exhibited sensory symptoms, and eight patients (patients 3–5, 7–10, 12) were severe cases, with symptoms including pain of burning quality, hyperalgesia, and allodynia (Fig. 4). Among the nine patients with SFN, four had length-dependent SFN (patients 3–5, 15) and five had non-length-dependent SFN/ganglionopathy (patients 7–9, 12, 14). Immunotherapy was effective in all patients except patient 14.

**Table 1:** Cases of neurosarcoidosis with severe autonomic dysfunction

Patient	Age (y)	Sex	Type of neuropathy^*^	Age at onset of sarcoidosis (y)	Age at onset of dysautonomia (y)	Interval between sarcoidosis and dysautonomia (y)	Sicca	OH/OI^*^	SwD^¶^	Upper GI^†^	Lower GI^$^	SeD^‡^	Dysuria^♮^	PA^§^	Sensory symptoms^#^	Complications	Immunotherapy and outcome
1^13^	31	F	CN + PRN	31	31	0	−	+	+	−	−	+	+	+	+	−	− : Improved
2^14^	49	F	CN + PRN	49	49	4 m	−	+	−	−	−	−	−	+	+	Hyperthyroidism, DM	PSL 40 mg/day: Improved
3^15^	71	F	SFN	67	67	0	−	+	+	−	+	−	+	−	+ +	−	− : Improved
4	67	F	SFN	43	64	21	−	−	+	−	+	−	−	−	+ +	−	− : Improved
5^16^	39	M	SFN	39	39	7 m	+	+	+	−	+	+	−	−	+ +	Type 2 DM	PSL 40 mg/day: No effect PSL 10 mg/day + MTX 20 mg: No effect IFX 3 mg/kg (500 mg) every 4 weeks and the dose tapered to 400 mg: Improved
6^17^	51	M	MM	51	51	0	+	+	−	+	+	+	−	−	+	Hyperthyroidism (Subacute thyroiditis)	PSL 40 mg/day: Improved
7^18^	27	F	SFN	26	27	1	+	+	−	−	−	−	−	−	+ +	−	IVIg (2 g/kg initially, then 1 g/kg after 2 weeks, then 1 g/kg every 4 weeks) + PSL + MTX: Improved
8	48	M	SFN	44	48	4	−	+	−	+	+	+	−	−	+ +	−	IVIg (2 g/kg initially, then 1 g/kg after 2 weeks, then maintenance doses of 1 g/kg every 4 weeks) + PSL + MTX + IFX: Improved
9	42	M	SFN	40	42	2	−	+	+	−	−	+	−	−	+ +	−	IVIg (2 g/kg initially, then 1 g/kg after 2 weeks, then maintenance doses of 1 g/kg every 4 weeks) + IFX: Improved
10^19^	67	M	CN + PRN	67	60	7	+	+	−	−	+	+	+	+	+ +	DM	PSL 50 mg/day: Improved
11^20^	38	F	SN	38	38	0	−	+	−	+	+	−	−	−	+	DM, bronchial asthma	IVIg: Improved
12^21^	59	F	SFN	59	59	0	−	+	−	−	+	−	+	−	+ +	−	IVMP + PSL 40 mg/day: Improved
13	64	M	AN	62	64	2	+	+	−	−	+	+	−	−	−	−	− : Improved
14	69	M	SFN	68	59	9	−	+	−	−	+	−	+	−	+	−	IVMP + PSL 40 mg/day with a gradual taper to 5 mg per day by 5 mg per day in 1 month + IVIg: No effect
15	27	M	SFN	27	27	0	+	+	+	−	+	+	+	N/A	+	Type 1 DM	PSL 60 mg/day in 1 month, followed by a gradual taper to 15 mg per day by 5 mg per day: Improved

^*^Patient 1 = Bilateral olfactory, trigeminal, facial, glossopharyngeal, vagus, accessory nerve palsy, 2 = facial nerve palsy, 4 = At the age of 44, she was diagnosed with central nervous system sarcoidosis due to ataxic gait, dysarthria, and headache. 6 = axonal sensory and motor polyneuropathy, 10 = facial, glossopharyngeal, vagus, and hypoglossal nerve palsy, axonal sensory and motor polyneuropathy, 11 = axonal sensory polyneuropathy

^*^Patient 7 = postural orthostatic tachycardia, 8 = inappropriate sinus tachycardia, 15 = neurally mediated syncope

^¶^Patient 1 = hypohidrosis of lower extremities, 3, 4, 15 = hypohidrosis, 5, 9 = hyperhidrosis

^†^Patient 6 = weight loss, 8 = nausea, 11 = weight loss, vomiting

Patient 3, 4, 10, 12, 13 = constipation, 5 = diarrhea, 6 = abdominal distension, constipation, 8 = constipation, gastroparesis, 11 = abdominal pain, gastroparesis, 14, 15 = alternating loose stools and constipation

^‡^Patient 1 = amenorrhea, 5, 6, 8, 9, 10, 13, 15 = impotence

^♮^Patient 1, 10, 12 = difficulty urinating, 3, 15 = frequent urination, 14 = urinary retention

^§^Patient 1 = anisocoria, 2 = Adie’s pupil, 10 = pseudo-Argyll Robertson pupil

^#^Patient 1 = peripheral hypoesthesia, 2 = truncal pain (C8-Th6 level), 3, 4, 7 = disabling peripheral pain with hyperalgesia, 5 = progressive burning sensations in both feet and hands, 8 = numbness, tingling, and disabling pain of the face, hands, legs, feet, and chest, 9 = severe burning pain associated with numbness and tingling of the hands, feet, forearms, legs, and chest, 10 = peripheral numbness, loss of vibration of lower extremities, 11 = peripheral sensory neuropathy, 12 = numbness in the limbs with allodynia in the back, 14 = burning sensation from the umbilicus to the lateral side of lower leg and great toe, 15 = paraesthesia in distal extremities

*AN* autonomic neuropathy, *CN* cranial neuropathy, *DM* diabetic mellitus, *F* female, *GI* gastrointestinal symptoms, *IFX* infliximab, *IVIg* intravenous injection of immunoglobulin, *IVMP* intravenous methylprednisolone pulse therapy, *M* male, *m* months, *MM* mononeuropathy multiplex, *MTX* methotrexate, *N/A* not available, *OH* orthostatic hypotension, *OI* orthostatic intolerance, *PA* pupillary abnormality, *PRN* polyradiculoneuropathy, *PSL* prednisolone, *SeD* sexual dysfunction, *SFN* small fiber neuropathy, *SN* sensory neuropathy, *SwD* sweating dysfunction

The comparison of the frequency of autonomic dysfunction in neurosarcoidosis with severe dysautonomia and anti-gAChR antibody-positive AAG is shown in Table 2. Orthostatic hypotension and orthostatic intolerance were most common in both groups, followed by lower gastrointestinal symptoms. Patients with neurosarcoidosis with dysautonomia were significantly younger at diagnosis than those with anti-gAChR antibody-positive AAG. Sexual dysfunction was significantly more frequent in neurosarcoidosis with dysautonomia than in anti-gAChR antibody-positive AAG.

**Table 2:** Characteristics of autonomic dysfunction in neurosarcoidosis with dysautonomia and anti-gAChR antibody-positive AAG

	Neurosarcoidosis with dysautonomia	Anti-gAChR antibody-positive AAG	P value
Number of patients	15	179	
Sex, number of male patients	8 (53.3%)	106 (59.0%)	.864
Age at diagnosis (years)	49.9 ± 15.1	59.0 ± 20.0	.031*
OH	14 (93.3%)	134 (74.9%)	.194
OI	14 (93.3%)	148 (82.7%)	.480
Arrhythmia	0 (0%)	32 (21.1%)	.153
Pupillary abnormalities	3 (20.0%)	16 (8.9%)	.351
Sicca	6 (40.0%)	81 (45.3%)	.902
Coughing	0 (0%)	25 (14.0%)	.250
Anhidrosis	6 (40.0%)	87 (48.6%)	.710
Upper GI dysfunction	3 (20.0%)	73 (40.8%)	.191
Lower GI dysfunction	11 (73.3%)	132 (73.7%)	.787
Bladder dysfunction	5 (33.3%)	101 (56.4%)	.146
Sexual dysfunction	8 (100.0%)	30 (28.3%)	< 0.001*

*AAG* autoimmune autonomic ganglionopathy, *gAChR* ganglionic acetylcholine receptor, *GI* gastrointestinal, *OH* orthostatic hypotension, *OI* orthostatic intolerance

Values are given as mean ± SD

P < 0.05 was considered statistically significant among the two groups (neurosarcoidosis with dysautonomia and anti-gAChR antibodies-positive AAG)

The laboratory findings of sarcoidosis for the 15 patients are shown in Table 3. Hypergammaglobulinemia was observed in seven (patients 3, 6, 10–13, 15) of nine patients. Anti-gAChR antibodies were present in two of three patients (patients 13, 14), of whom one was affected by probable SFN and the other by autonomic neuropathy.

**Table 3:** Laboratory findings of cases of neurosarcoidosis with severe autonomic dysfunction

Patient	Age	Sex	BHL	Serum IgG (mg/dl)	ACE (U/L)	Lysozyme (μg/ml)	sIL-2R (U/ml)	Serum Ca (mg/dl)	Tuberculin skin test	BALF CD4/CD8 ratios	Cell count of CSF (/μl)	Protein level of CSF (mg/dl)	Ga^67^ scintigraphy^#^	NCS^$^	Sural nerve biopsy^§^	Skin biopsy^*^ (IENFD)	Anti-gAChR antibody
1^13^	31	F	+	N/A	21.6	18.4	N/A	N/A	−	N/A	2	85	Abnormal	W. N. L	W. N. L	N/A	N/A
2^14^	49	F	−	N/A	66.0	High	N/A	N/A	−	N/A	High	High	Abnormal	W. N. L	N/A	N/A	N/A
3^15^	71	F	+	1,721	9.4	N/A	N/A	9.5	N/A	N/A	N/A	N/A	Abnormal	W. N. L	N/A	Abnormal	N/A
4	67	F	+	626	9.7	N/A	N/A	10	N/A	N/A	N/A	N/A	N/A	W. N. L	N/A	Abnormal	N/A
5^16^	39	M	+	N/A	22.0	N/A	725	N/A	N/A	N/A	N/A	N/A	N/A	W. N. L	N/A	Abnormal	N/A
6^17^	51	M	+	1,374	20.6	18.5	N/A	10.5	N/A	N/A	3.9	104	Abnormal	Abnormal	Abnormal	N/A	N/A
7^18^	27	F	+	W. N. L	N/A	N/A	N/A	N/A	N/A	N/A	N/A	N/A	N/A	W. N. L	N/A	Abnormal	N/A
8	48	M	+	W. N. L	N/A	N/A	N/A	N/A	N/A	N/A	N/A	N/A	N/A	W. N. L	N/A	Abnormal	N/A
9	42	M	+	W. N. L	N/A	N/A	N/A	N/A	N/A	N/A	N/A	N/A	N/A	W. N. L	N/A	Abnormal	N/A
10^19^	67	M	+	2,448	W. N. L	W. N. L	2,153	W. N. L	−	N/A	< 1	45	Abnormal	Abnormal	Abnormal	N/A	N/A
11^20^	38	F	−	2,800	N/A	N/A	N/A	N/A	N/A	N/A	N/A	N/A	−	Abnormal	Abnormal	N/A	−
12^21^	59	F	+	2,009	52	20.6	3,430	9.9	−	3.37	High	High	Abnormal	W. N. L	N/A	Abnormal	N/A
13	64	M	N/A	1,345.5	40.4	N/A	N/A	9.5	N/A	N/A	1/3	44	N/A	W. N. L	N/A	N/A	+
14	69	M	+	1,145	22.8	N/A	684	9.1	N/A	+	1	37	Abnormal	W. N. L	Abnormal	Abnormal	+
15	27	M	+	1,466	17.2	N/A	763	9.5	−	N/A	4	52	Abnormal	W. N. L	N/A	N/A	−

^#^Patient 1 = accumulations in hilar lymph node, lacrimal and parotid gland, 2 = accumulations in subcutaneous mass, 3 = accumulations in hilar and mediastinal lymph nodes, 6 = accumulations in bilateral lacrimal, parotid and submandibular gland, hilar and mediastinal lymph nodes, and left subclavian lymph nodes, 10 = accumulations in bilateral hilar, mediastinal and inguinal lymph nodes, and parotid gland, 11 = no accumulations in PET scan, 12 = accumulations in hilar and mediastinal lymph nodes, 14 = accumulations in bilateral hilar, mediastinal and subclavian lymph nodes in PET scan, 15 = accumulations in lacrimal and salivary glands, para-aortic and left groin lymph nodes, and left testis

^$^Patient 6 = decreased amplitude of CMAP and MCV in median nerve, not evoked of SNAP in upper and lower extremities, prolonged latency of bilateral N9 and N37 in SEP, 10 = severe decreased amplitude of CMAP and MCV, not evoked of SNAP in the upper and lower extremities, prolonged F wave conduction velocity, prolonged latency between N9 and 13 in SEP, 11 = axonal length-dependent neuropathy

^§^Patient 6 = vasculitis in epineurium, decreased unmyelinated nerve in electronic microscope and axonal degeneration and increase of collagen pocket in teased-fiber preparation, 10 = severe decreased myelinated and unmyelinated nerve density and myelin ovoid and thinning of myelinated fiber, 11 = epithelioid cell granuloma and denervation in neuromuscular biopsy, 14 = mild lymph cell invasion around small vessel of epineurium

^*^Patient 15 = dermal nerve fiber density

*ACE* Angiotensin-converting enzyme, *BALF* bronchoalveolar lavage fluid, *BHL* bilateral hilar lymphadenopathy, *Ca* calcium, *CMAP* compound muscle action potential, *CSF* cerebrospinal fluid, *Ga*^*67*^ Gallium-67, *gAChR* ganglionic acetylcholine receptor, *IENFD* intra-epidermal nerve fiber density, *MCV* motor conduction velocity, *N/A* not available, *NCS* nerve conduction study, *PET* positron emission tomography, *SEP* sensory-evoked potential, *sIL-2R* soluble interleukin-2 receptor, *SNAP* sensory nerve action potential, *W. N. L* within normal limits

The autonomic function test results for neurosarcoidosis with severe dysautonomia in six (patients 1, 6, 10, 13–15) of 15 patients who had undergone detailed examination are shown in Table 4. Patient 1 had a preganglionic disorder in the sympathetic nervous system, which was based on no response to 5% eye-drop solution of cocaine, a normal intradermal injection of acetylcholine despite reduced sweating in lower extremities, and increased plasma noradrenaline responsiveness upon postural change from supine to standing position. In the parasympathetic nervous system, the absence of supersensitivity to a 2.5% eye-drop solution of methacholine indicated no postganglionic impairment. Patient 6 had a postganglionic disorder in the sympathetic nervous system, which was based on no increase in plasma noradrenaline responsiveness upon postural change from supine to standing position, supersensitivity to a 1.25% eye-drop solution of adrenaline, and the absence of sympathetic skin response. In Patient 10, mydriasis and attenuated mydriasis were observed upon eye-drop solution of 5% tyramine and 5% cocaine administration, respectively, indicating preganglionic disturbance in the sympathetic nervous system. In addition, elevation in plasma noradrenaline concentration upon postural change from supine to standing position and the normal H/M ratio by MIBG myocardial scintigraphy suggested no sympathetic postganglionic impairment. In the parasympathetic nervous system, the absence of supersensitivity to eye-drop solution of 0.25% pilocarpine indicated no postganglionic impairment. Decreased sensation and voluntary inability to urinate, a large amount of residual urine, low bladder pressure, and atonic and involuntary pattern of contraction in bladder functional test indicated autonomous bladder. Spinal cord MRI showed no cauda equina lesions, suggesting parasympathetic preganglionic impairment. Patients 13 and 15 had postganglionic disorders in the sympathetic nervous system based on a reduced H/M ratio in ^123^I-MIBG myocardial scintigraphy. Patient 14 also had postganglionic disorders in the sympathetic nervous system based on a reduced H/M ratio in ^123^I-MIBG myocardial scintigraphy and low amplitude of sympathetic skin response. Patients 2 and 9 had postganglionic disorders based on Adie’s pupil and abnormality in the quantitative sudomotor axon reflex test, respectively (data not shown in Table 4).

**Table 4:** Autonomic function test in neurosarcoidosis with severe autonomic dysfunction

Patient		1^13^	6^17^	10^19^	13	14	15
Age (years)		31	51	67	64	69	27
Site of lesion in dysautonomia		Preganglionic	Postganglionic	Preganglionic	Postganglionic	Postganglionic	Postganglionic
Clinical form		CN + PRN	MM	CN + PRN	AN	SFN	SFN
*Eye drop test (pupillary responses to local instillation)*							
5% cocaine		NR	NR	Slightly pupil dilates	N.D	N.D	N.D
Supersensitivity to 1.25% adrenaline		−	+	N.D	N.D	N.D	N.D
1% phenylephrine		N.D	N.D	Normal	N.D	N.D	N.D
5% tyramine		Normal	N.D	Normal	N.D	N.D	N.D
Supersensitivity to 2.5% methacholine		−	−	N.D	N.D	N.D	N.D
Supersensitivity to 0.1% pilocarpine^*^		−	N.D	−	N.D	N.D	N.D
*Sudomotor and cutaneous vasomotor test*							
Thermoregulatory sweat test		Reduced sweating in lower extremities	N.D	N.D	N.D	N.D	Reduced
ACh sweat test (5% acetylcholine)		Normal	Normal	N.D	N.D	N.D	N.D
Sympathetic skin response		N.D	NR	N.D	N.D	Decreased	N.D
*Cardiovascular function test*							
Head up tilt test	BP response to postural change, BP (mmHg)	126/82 → 85/48 (45° angle of tilt for 3 min, nausea occurred) 119/82 → 67/31 (90° angle of tilt for 7 min, syncope occurred)	138/98 → Unmeasurable (1 min) → 103/62 (2 min) → 120/72 (5 min)	114/68 → 49/36 (5 min) → 54/39 (10 min)	N.D	N.D	100/75 → 80/49 (70° angle of tilt for 20 min under isoproterenol provocation)
	HR response to postural change, HR (/min)	99 → 117 (45° angle of tilt for 3 min) 101 → 121 (90° angle of tilt for 7 min)	73 → Unmeasurable (1 min) → 90 (2 min) → 91 (5 min)	80 → 98 (5 min) → 98 (10 min)	N.D	N.D	89 → 120 (70° angle of tilt for 20 min)
	Plasma NA change from supine to standing position (pg/mL) (100 < Normal < 450)	W. N. L 250 → 360 (45° angle of tilt for 3 min)	W. N. L 70 → 120 (5 min)	W. N. L 210 → 440 (10 min)	N.D	N.D	W.N.L 145 → 398 (70° angle of tilt 5 min)
Cold pressor test (4 °C, 1 min)	BP response (mmHg)	132/98 → 142/100	131/80 → 142/84	N.D	N.D	N.D	N.D
Supersensitivity to NA infusion (0.05–0.10 μg/kg, 6 min)		−	+	N.D	N.D	N.D	N.D
Reduced CV R–R (%)		+	+	+	+	+	+
Myocardial ^123^I-MIBG scintigraphy, H/M ratio (early)		N.D	N.D	Normal	Decreased	Decreased	Decreased
Myocardial ^123^I-MIBG scintigraphy, H/M ratio (delayed)		N.D	N.D	Normal	Decreased	Decreased	Decreased
*Urodynamic studies*							
Decreased urinary flow		N.D	N.D	+	N.D	N.D	N.D
Post-void residual urine (mL) (Normal < 30)		N.D	N.D	400	N.D	N.D	N.D
Bladder capacity (mL) (200 < Normal < 600)		N.D	N.D	> 500	N.D	N.D	N.D
Maximum urethral closure pressure (cm H_2_O) (41 < Normal < 82)		N.D	N.D	44	N.D	N.D	N.D
Detrusor areflexia on voiding		N.D	N.D	+	N.D	N.D	N.D

*ACh* acetylcholine, *AN* autonomic neuropathy, *BP* blood pressure, *CN* cranial neuropathy, *PRN* polyradiculoneuropathy, CV R–R coefficient of variation in R–R, *H/M ratio* heart-to-mediastinum ratio, *NA* noradrenaline, *N.D.* not done, *NR* no response, *MIBG* metaiodobenzylguanidine, *MM* mononeuropathy multiplex, *PRN* polyradiculoneuropathy, *SFN* small fiber neuropathy, *W.N.L* within normal limits

^*^Patient 10 = 0.25% pilocarpine

## Discussion

We described three cases of neurosarcoidosis with dysautonomia, including anti-gAChR-positive cases. We also systematically reviewed the literature regarding neurosarcoidosis with dysautonomia.

Comparison of the frequency of autonomic dysfunction among patients with neurosarcoidosis with severe dysautonomia and those with anti-gAChR antibody-positive AAG revealed that orthostatic hypotension and orthostatic intolerance, followed by gastrointestinal symptoms, were the most common presentations in both groups. These results in neurosarcoidosis with severe dysautonomia are similar to those reported previously [22]. Further, they support similar autonomic symptoms’ characteristics between neurosarcoidosis with autonomic dysfunction and AAG.

Hypergammaglobulinemia was observed in seven of nine patients. Hypergammaglobulinemia was previously reported in patients with sarcoidosis [23]. Moreover, it has been suggested that hypergammaglobulinemia or circulating immune complexes may be associated with the clinical course of sarcoidosis [23, 24]. A previous study also reported the presence of higher levels of B-cell activating factor, and correspondingly, higher numbers of IgG-producing B cells in the lung tissue [25]. The imbalance in the number of B cells and T follicular helper cell subsets is particularly important for antibody production and may be associated with development of sarcoidosis [26]. Patients with sarcoidosis frequently have higher titers of autoantibodies than healthy individuals [27–29]. Several autoantibodies, including anti-mitochondrial, anti-nuclear, anti-double stranded DNA, anti-citrullinated cyclic peptide, rheumatoid factor, anti-vimentin, and anti-negative elongation factor E, have been detected in sarcoidosis [27–34]. To the best of our knowledge, no previous study has reported anti-gAChR antibodies in sarcoidosis.

Among the presented cases and those in the reviewed literature, the most common type of neuropathy was SFN. There are two categories of SFN clinical symptoms: sensory symptoms such as neuropathic pain, allodynia, hyperalgesia, or pinprick and thermal sensory loss, and autonomic symptoms, including orthostatic intolerance, sicca symptoms, sweating disturbances, constipation, urinary disturbance, and sexual dysfunction. According to the self-reported burden, sarcoid-associated SFN (SSFN) was reported to be present in 86.2% of patients with sarcoidosis but may be overlooked by clinicians [35]. Patients with sarcoidosis with symptoms of SFN were found to have significantly decreased IENFDs as compared to patients with asymptomatic sarcoidosis and healthy individuals [36]. In our literature review, we focused on sarcoidosis with dysautonomia and found that many patients also have sensory symptoms, among whom seven patients were classified as definite SFN. Moreover, five were classified as non-length dependent SFN, one of whom was positive for anti-gAChR antibodies. Non-length dependent SFN is more commonly observed in immune-mediated conditions than length-dependent SFN, and three IVIg-responsive cases of sarcoidosis affected by non-length-dependent SFN have been described [37]. We previously reported a 46% rate of sensory disturbance among patients with anti-gAChR antibody-positive AAG [38], while a post-mortem study reported one case of a patient with non-length-dependent SFN with nicotinic AChR antibodies [39]. The pathological mechanism of SSFN is known to involve proinflammatory cytokines, including tumor necrosis factor-α [16]. It was reported that patients with sarcoidosis carrying the HLA-DQB1*0602 allele are more likely to have abnormal temperature threshold testing, indicative of SFN, than do healthy individuals [40]. Although this result simply reflects a bystander effect in the pathogenesis of sarcoidosis, our cases suggest that anti-gAChR antibodies may contribute to the pathogenesis of SSFN.

At the site of lesion in dysautonomia, postganglionic denervation was more common, and we found reduced H/M ratios and increased washout rates in ^123^I-MIBG myocardial scintigraphy in our three illustrative cases. Cardiac sympathetic dysfunction in sarcoidosis comorbid with SFN was previously demonstrated by ^123^I-MIBG myocardial scintigraphy [41]. Previous studies have revealed the presence of cardiac sympathetic dysfunction in up to 50% of patients with SSFN by ^123^I-MIBG myocardial scintigraphy [41, 42]. Importantly, anti-gAChR antibodies were detected in Cases 1 and 2 in our study. It has been demonstrated that a decreased H/M ratio is observed in 80% of seropositive AAG cases, with several cases showing improvements in their H/M ratio by immunotherapy [8, 38]. In the present Case 3, the reduced H/M ratio in^123^I-MIBG myocardial scintigraphy was improved by steroid therapy; therefore, it may have resulted from functional impairments in autonomic nerve fiber transmission. Autoantibodies against gAChR, which could have interfered with autonomic ganglionic synaptic transmission of the sympathetic and parasympathetic nerves, were not detected in this case. We speculate that other immunologic blocking factors, such as unknown antibodies or cytokines, may have caused the functional impairment in the postganglionic pathways.

Two patients (patients 1 and 10) presented with preganglionic autonomic dysfunction. Although it is not possible to determine whether the site of lesions in dysautonomia is the preganglionic fiber or the central nervous system strictly based on the results of the autonomic function test, we considered that the site of lesion of autonomic disturbance may be the spinal nerve root level. This was based on the fact that there was no loss of myelinated and unmyelinated fibers in sural nerve biopsy and that the levels of cerebrospinal fluid protein were elevated in patient 1, while the F wave conduction velocities in nerve conduction studies were prolonged in patient 10. Interestingly, both these patients had cranial neuropathy with polyradiculoneuropathy. We also consider that the preganglionic fiber disorder might have occurred as a partial symptom of sarcoid polyradiculoneuropathy. Regarding the distribution of sensory disturbance in the 15 literature cases, there were many cases in which peripheral nerves, including small fibers, were the site of lesion; however, in three cases of cranial neuropathy with polyradiculoneuropathy, the lesion site may have been the dorsal root. Eleven patients underwent immunotherapy, all of whom, except one (Case 2), were responsive to immunotherapy. While some patients with sarcoidosis-related autonomic dysfunction showed improvements after treatment with oral prednisolone alone, there were several intractable cases that were only improved after combining multiple therapeutic agents, such as intravenous methylprednisolone pulse, IVIg, prednisolone, and immunosuppressants. The seropositive patient (Case 2) did not show any improvement despite immunotherapy by steroid pulse, oral prednisolone, and IVIg. Past studies have suggested the use of a combined therapy consisting of steroids, plasmapheresis, or IVIg, followed by oral prednisolone and immunosuppressive agents, such as azathioprine, mycophenolate mofetil, and rituximab, in severe patients with AAG [8]. Therefore, we speculated that AAG might be occurring in combination with neurosarcoidosis in this case, and that a combination of immunotherapy other than steroids and IVIg may be necessary.

This study has several limitations. First, we could not follow-up on the patients after the treatment for potential recurrences. Second, five of the 15 cases had diabetes mellitus, which may have affected autonomic dysfunction. Based on the dramatic response to immunotherapy, we surmised that these cases of autonomic dysfunction may be caused by dysimmune mechanisms related to sarcoidosis, although coexisting underlying diabetic neuropathy cannot be excluded. Third, the present study included a small study population without appropriate control groups. Further, we could not confirm whether anti-gAChR antibodies were present in patients with normal sarcoidosis or neurosarcoidosis without autonomic dysfunction. Therefore, we plan to assess this in our future studies. Finally, we previously reported that the levels of anti-gAChR antibodies may not necessarily affect the severity of the disease among patients with anti-gAChR-positive AAG [12]. Further prospective studies involving a large number of patients are necessary to address the prevalence of anti-gAChR antibodies among patients with neurosarcoidosis with dysautonomia, assess the rates of responsiveness to immunotherapy depending on the presence or absence of anti-gAChR antibodies, and investigate potential treatment strategies.

## Conclusion

We reported neurosarcoidosis cases with severe dysautonomia, cases that have tested positive for anti-gAChR antibodies. Anti-gAChR antibodies may be associated with the pathogenesis of dysautonomia in sarcoidosis. Further studies are needed to elucidate the relationship between neurosarcoidosis with dysautonomia and anti-gAChR antibodies, and prospective studies to investigate potential treatment strategies are warranted.

## Data Availability

The data that support the findings of this study are available from the corresponding author upon reasonable request.

## References

[1] Delaney P (1977). Neurologic manifestations in sarcoidosis: review of the literature, with a report of 23 cases. Ann Intern Med.

[2] Lower EE, Broderick JP, Brott TG, Baughman RR (1997). Diagnosis and management of neurological sarcoidosis. Arch Intern Med.

[3] Hoitsma E, Faber CG, Drent M, Sharma OP (2004). Neurosarcoidosis: a clinical dilemma. Lancet Neurol.

[4] Hoitsma E, Marziniak M, Faber CG, Reulen JPH, Sommer C, De Baets M, Drent M (2002). Small fibre neuropathy in sarcoidosis. Lancet.

[5] Tavee J (2018). Office approach to small fiber neuropathy. Cleve Clin J Med.

[6] Tavee J, Culver D (2019). Non-organ manifestations of sarcoidosis. Curr Opin Pulm Med.

[7] Wang Z, Low PA, Vernino S (2010). Antibody-mediated impairment and homeostatic plasticity of autonomic ganglionic synaptic transmission. Exp Neurol.

[8] Nakane S, Higuchi O, Koga M, Kanda T, Murata K, Suzuki T, Kurono H, Kunimoto M, Kaida K, Mukaino A, Sakai W, Maeda Y, Matsuo H (2015). Clinical features of autoimmune autonomic ganglionopathy and the detection of subunit-specific autoantibodies to the ganglionic acetylcholine receptor in Japanese patients. PLoS ONE.

[9] Hunninghake GW, Costabel U, Ando M, Baughman R, Cordier JF, du Bois R, Eklund A, Kitaichi M, Lynch J, Rizzato G, Rose C, Selroos O, Semenzato G, Sharma OP (1999). ATS/ERS/WASOG statement on sarcoidosis. American Thoracic Society/European Respiratory Society/World Association of Sarcoidosis and other Granulomatous Disorders. Sarcoidosis Vasc Diffuse Lung Dis.

[10] Lacomis D (2002). Small-fiber neuropathy. Muscle Nerve.

[11] Moher D, Liberati A, Tetzlaff J, Altman DG; PRISMA Group (2009). Preferred reporting items for systematic reviews and meta-analyses: the PRISMA statement. PLoS Med.

[12] Nakane S, Mukaino A, Higuchi O, Maeda Y, Takamatsu K, Yamakawa M, Watari M, Tawara N, Nakahara K, Kawakami A, Matsuo H, Ando Y (2020). A comprehensive analysis of the clinical characteristics and laboratory features in 179 patients with autoimmune autonomic ganglionopathy. J Autoimmun.

[13] Kazahaya Y, Kita K, Nakano Y, Hirayama K (1985). Sarcoid polyradiculoneuropathy with autonomic dysfunctions. Rinsho Shinkeigaku.

[14] Otsuka H, Morikawa N, Mukae H et al (1987) A case of sarcoidosis with various neurological symptoms, discovered with subcutaneous nodules. Nihon Sarcoidosis/Nikugeseishikkan Gakkaizasshi (The Japanese Journal of Sarcoidosis and Other Granulomatous Disorders) 7:69–70 (in Japanese)

[15] Ikeda Y, Yamaguchi T, Yamada Y, Shinohara T, Kono C, Aoyagi T, Amano H, Kijima M, Kurose N, Miyamoto K (2004) Two sarcoidosis cases of small fiber neuropathy. Nihon sarcoidosis/nikugeseishikkan gakkaizasshi (The Japanese Journal of Sarcoidosis and Other Granulomatous Disorders) 24:65–69 (in Japanese)

[16] Hoitsma E, Faber CG, van Santen-Hoeufft M, De Vries J, Reulen JP, Drent M (2006). Improvement of small fiber neuropathy in a sarcoidosis patient after treatment with infliximab. Sarcoidosis Vasc Diffuse Lung Dis.

[17] Kimura Y, Takeuchi M, Ota K, Uchiyama S, Iwata M (2007) A case of sarcoidosis with severe autonomic dysfunction. Tokyo Jyoshi Ika Daigaku zasshi suppl:99–104.

[18] Parambil JG, Tavee JO, Zhou L, Pearson KS, Culver DA (2011). Efficacy of intravenous immunoglobulin for small fiber neuropathy associated with sarcoidosis. Respir Med.

[19] Yabuuchi K, Okazaki T, Nakamura K, Hanaoka T, Kimura N, Mori T, Hirano T, Kumamoto T (2013) A male case aged 67 of neurosarcoidosis with severe autonomic dysfunction. Nihon Sarcoidosis/Nikugeseishikkan Gakkaizasshi (The Japanese Journal of Sarcoidosis and Other Granulomatous Disorders) 33:139–145 (in Japanese)

[20] Grignano E, Mekinian A, Dubourg O, Dhote R, Fain O (2013). Dramatic response to intravenous immunoglobulins in dysautonomic neurosarcoidosis. J Clin Neurosci.

[21] Saito H, Yamaguchi T, Adachi Y, Yamashita T, Wakai Y, Saito K, Shinohara Y, Suzuki K, Yagihashi S, Terada J, Tatsumi K (2015). Neurological symptoms of sarcoidosis-induced small fiber neuropathy effectively relieved with high-dose steroid pulse therapy. Intern Med.

[22] Tavee JO, Karwa K, Ahmed Z, Thompson N, Parambil J, Culver DA (2017). Sarcoidosis-associated small fiber neuropathy in a large cohort: Clinical aspects and response to IVIG and anti-TNF alpha treatment. Respir Med.

[23] Kataria YP, Holter JF (1997). Immunology of sarcoidosis. Clin Chest Med.

[24] Rømer FK, Sølling J (1981). Relationship between circulating immune complexes and angiotensin-converting enzyme in pulmonary sarcoidosis. Acta Med Scand.

[25] Saussine A, Tazi A, Feuillet S, Rybojad M, Juillard C, Bergeron A, Dessirier V, Bouhidel F, Janin A, Bensussan A, Bagot M, Bouaziz JD (2012). Active chronic sarcoidosis is characterized by increased transitional blood B cells, increased IL-10-producing regulatory B cells and high BAFF levels. PLoS ONE.

[26] Kudryavtsev I, Serebriakova M, Starshinova A (2020). Imbalance in B cell and T follicular helper cell subsets in pulmonary sarcoidosis. Sci Rep.

[27] Weinberg I, Vasiliev L, Gotsman I (2000). Anti-dsDNA antibodies in sarcoidosis. Semin Arthritis Rheum.

[28] Kobak S, Yilmaz H, Sever F, Duran A, Sen N, Karaarslan A (2014). The prevalence of antinuclear antibodies in patients with sarcoidosis. Autoimmune Dis.

[29] Kobak S, Ylmaz H, Sever F, Duran A, Sen N (2014). Anti-cyclic citrullinated peptide antibodies in patients with sarcoidosis. Sarcoidosis Vasc Diffuse Lung Dis.

[30] Maddrey WC, Johns CJ, Boitnott JK, Iber FL (1970). Sarcoidosis and chronic hepatic disease: a clinical and pathologic study of 20 patients. Medicine (Baltimore).

[31] Fagan EA, Moore-Gillon JC, Turner-Warwick M (1983). Multiorgan granulomas and mitochondrial antibodies. N Engl J Med.

[32] Stanca CM, Fiel MI, Allina J, Caracta CF, Odin JA (2005) Liver failure in an anti-mitochondrial antibody-positive patient with sarcoidosis: primary biliary cirrhosis or hepatic sarcoidosis? Semin Liver Dis 25:364–370. https://doi.org/10.1055/s-2005-916327 10.1055/s-2005-916327 16143951

[33] Kinloch AJ, Kaiser Y, Wolfgeher AJ, Eklund A, Clark MR, Grunewald J (2018). In situ humoral immunity to vimentin in HLA-DRB1*03^+^ patients with pulmonary sarcoidosis. Front Immunol.

[34] Baerlecken N, Pursche N, Witte T, Kniesch K, Höpfner M, Ernst D, Moosig F, Seeliger B, Prasse A (2020). Presence of antibodies binding to negative elongation factor E in sarcoidosis. J Clin Med.

[35] Voortman M, Hendriks CMR, Elfferich MDP, Bonella F, Møller J, De Vries J, Costabel U, Drent M (2019). The burden of sarcoidosis symptoms from a patient perspective. Lung.

[36] Bakkers M, Merkies IS, Lauria G, Devigili G, Penza P, Lombardi R, Hermans MC, van Nes SI, De Baets M (2009). Faber CG (2009) Intra-epidermal nerve fiber density and its application in sarcoidosis. Neurology.

[37] Khan S, Zhou L (2012). Characterization of non-length-dependent small-fiber sensory neuropathy. Muscle Nerve.

[38] Nakane S, Mukaino A, Maeda Y, Higuchi O, Matsuo H, Ando Y (2017). Extra-autonomic manifestations in autoimmune autonomic ganglionopathy: a Japanese survey. J Neurol Neurosurg Psychiatry.

[39] Younger DS (2019). A postmortem study of a patient with low titer nicotinic acetylcholine receptor ganglionic antibody: implications for clinical neurologic disease. World J Neurosci.

[40] Voorter CE, Drent M, Hoitsma E, Faber KG, van den Berg-Loonen EM (2005). Association of HLA DQB1 0602 in sarcoidosis patients with small fiber neuropathy. Sarcoidosis Vasc Diffuse Lung Dis.

[41] Hoitsma E, Faber CG, van Kroonenburgh MJ, Gorgels AP, Halders SG, Heidendal GA, Kessels AG, Reulen JP, Drent M (2005). Association of small fiber neuropathy with cardiac sympathetic dysfunction in sarcoidosis. Sarcoidosis Vasc Diffuse Lung Dis.

[42] Smulders NM, Bast A, van Kroonenburgh MJ, Drent M (2008). Improvement of cardiac sympathetic nerve function in sarcoidosis. Sarcoidosis Vasc Diffuse Lung Dis.

